# Application of biomedical materials in the diagnosis and treatment of myocardial infarction

**DOI:** 10.1186/s12951-023-02063-2

**Published:** 2023-08-26

**Authors:** Jiahui Zhang, Yishan Guo, Yu Bai, Yumiao Wei

**Affiliations:** 1 Department of Cardiology, Union Hospital, Tongji Medical College, Huazhong University of Science and Technology, Wuhan, 430022 China; 2 Hubei Key Laboratory of Biological Targeted Therapy, Union Hospital, Tongji Medical College, Huazhong University of Science and Technology, Wuhan, 430022 China; 3 Hubei Engineering Research Center for Immunological Diagnosis and Therapy of Cardiovascular Diseases, Union Hospital, Tongji Medical College, Huazhong University of Science and Technology, Wuhan, 430022 China; 4 Department of Cardiology, Binzhou Medical University Hospital, Binzhou, 256600 China; 5 Graduate School, Peking Union Medical College, Chinese Academy of Medical Sciences, Beijing, 100000 China; 6 National Center for Respiratory Medicine; State Key Laboratory of Respiratory Health and Multimorbidity; National Clinical Research Center for Respiratory Diseases; Institute of Respiratory Medicine, Chinese Academy of Medical Sciences; Department of Pulmonary and Critical Care Medicine, Center of Respiratory Medicine, China-Japan Friendship Hospital, Beijing, P.R. China

**Keywords:** Biomedical materials, MI, Cardiac patch, Hydrogel, Nano biomaterial, Artificial blood vessel

## Abstract

Myocardial infarction (MI) is a cardiovascular emergency and the leading cause of death worldwide. Inflammatory and immune responses are initiated immediately after MI, leading to myocardial death, scarring, and ventricular remodeling. Current therapeutic approaches emphasize early restoration of ischemic myocardial reperfusion, but there is no effective treatment for the pathological changes of infarction. Biomedical materials development has brought new hope for MI diagnosis and treatment. Biomedical materials, such as cardiac patches, hydrogels, nano biomaterials, and artificial blood vessels, have played an irreplaceable role in MI diagnosis and treatment. They improve the accuracy and efficacy of MI diagnosis and offer further possibilities for reducing inflammation, immunomodulation, inhibiting fibrosis, and cardiac regeneration. This review focuses on the advances in biomedical materials applications in MI diagnosis and treatment. The current studies are outlined in terms of mechanisms of action and effects. It is addressed how biomedical materials application can lessen myocardial damage, encourage angiogenesis, and enhance heart function. Their clinical transformation value and application prospect are discussed.

## Introduction

Cardiovascular disease (CVD) has always been a major threat to human health. According to a World Health Organization report, heart diseases have been the leading cause of death globally for the past two decades. Since 2000, heart disease deaths have increased by more than two million and by approximately nine million in 2019, resulting in 17.9 million deaths, accounting for 32% of all deaths worldwide [1]. Myocardial infarction (MI) is the leading cause of death from all CVD. Numerous known risk factors of MI include smoking, high blood pressure, dyslipidemia, diabetes, obesity, unhealthy diet, and lack of exercise, significantly affecting life quality [2]. According to clinical classification, MI is divided into five types: spontaneous MI (types 1, 2, and 3) and peri-procedural MI (types 4 and 5) [3, 4]. According to electrocardiogram (ECG) diagnosis, MI is divided into ST-segment elevation myocardial infarction (STEMI) and non-ST-segment elevation myocardial infarction (NSTEMI) [5]. Each exhibits symptoms, including abrupt chest pain, dyspnea, and left arm pain. Severe MI may result in sudden cardiac death [6]. Therefore, prompt diagnosis and treatment significantly influence MI prognosis.

Currently, the main criteria to diagnose MI include clinical symptoms, the detection of cardiac biomarkers, and imaging tests. Regarding treatment, it is crucial to reperfuse the occluded vessel as soon as possible [7]. Thrombolytic agents can convert endogenous plasminogen into plasmin to promote fibrinolysis [8]. Thrombolytic drugs are recommended 6 to 12 h after MI [9]. Common thrombolytic agents include streptokinase, tissue plasminogen activator (tPA), and its recombinant forms (alteplase, reteplase, and tenecteplase) [10, 11]. Presently, surgical reperfusion is the preferred method in the clinic. Percutaneous coronary intervention (PCI) and coronary artery bypass grafting (CABG) are two common surgical procedures. A guidewire is inserted into a blood vessel to reach the coronary arteries during PCI, performed through an artery in the leg or arm. Then, reperfusion is achieved by balloon dilation and stent implantation [12]. CABG is typically performed when PCI is ineffective or with multivessel disease [10]. Reperfusion is achieved by implanting a vessel that allows blood to flow across the occluded vessel directly through the aorta to the infarcted area [13]. However, PCI and CABG have several disadvantages. The occurrence of restenosis limits PCI after stent implantation [14]. The drug-eluting stents can alleviate this problem to some extent [15]. CABG is an expensive procedure with severe postoperative complications, such as atrial fibrillation, thromboembolism, infection, renal injury, and neurological symptoms [16–20]. The mortality rate of MI has been reduced by 50–70% through these measures, but approximately 6% of patients with severe complications, such as cardiogenic shock, still exist [21]. Current treatment measures emphasize early and timely intervention to achieve reperfusion and reduce myocardial injury and ventricular remodeling, but there is no effective cardiac regeneration method for the infarcted myocardium [22]. In addition, it seems that active intervention can also reduce myocardial damage for the inflammatory response in the early stage of MI [23, 24]. Therefore, only restoring the blood supply in MI patients may be insufficient to restore cardiac function. It is necessary to fully restore cardiac function by reducing inflammation and scar repair while promoting angiogenesis and cell proliferation.

Biomedical materials are indispensable in modern medicine as a medium for implanting into the human body. Biomedical materials are classified according to their chemical structure (metals, ceramics, polymers, and composites), degree of interaction with the biological environment (inert, bioactive, and bioabsorbable), and origin (synthetic or natural) [25]. Biomedical materials can be loaded with bioactive substances such as cytokines, drugs, and cells, unlike sutures, gauze, or staples. Smart biomedical materials can also be activated under specific stimuli [26]. Biomedical materials can effectively diagnose and treat MI. Biomedical materials’ signal amplification and the porous structure can make detection specific and sensitive in diagnosis. Regarding treatment, biomedical materials’ physical and chemical properties have certain therapeutic effects. Moreover, biomedical materials can target the therapeutic site and form a scaffold to stably retain the cargo on the target, providing precise and efficient therapeutic effects [27]. However, biomaterial applications still face some difficulties. Porosity may affect the foreign body response after implantation [28]. More research is necessary to prove whether biomedical materials’ degradation rate and toxicity may have adverse effects [29]. Additionally, the mechanical properties of biomedical materials must be better tailored to the heart’s physiological functions due to the heart’s regular beating and pumping function. The delivery efficiency of biologically active substances also must be guaranteed [30].

**Fig. 1:**
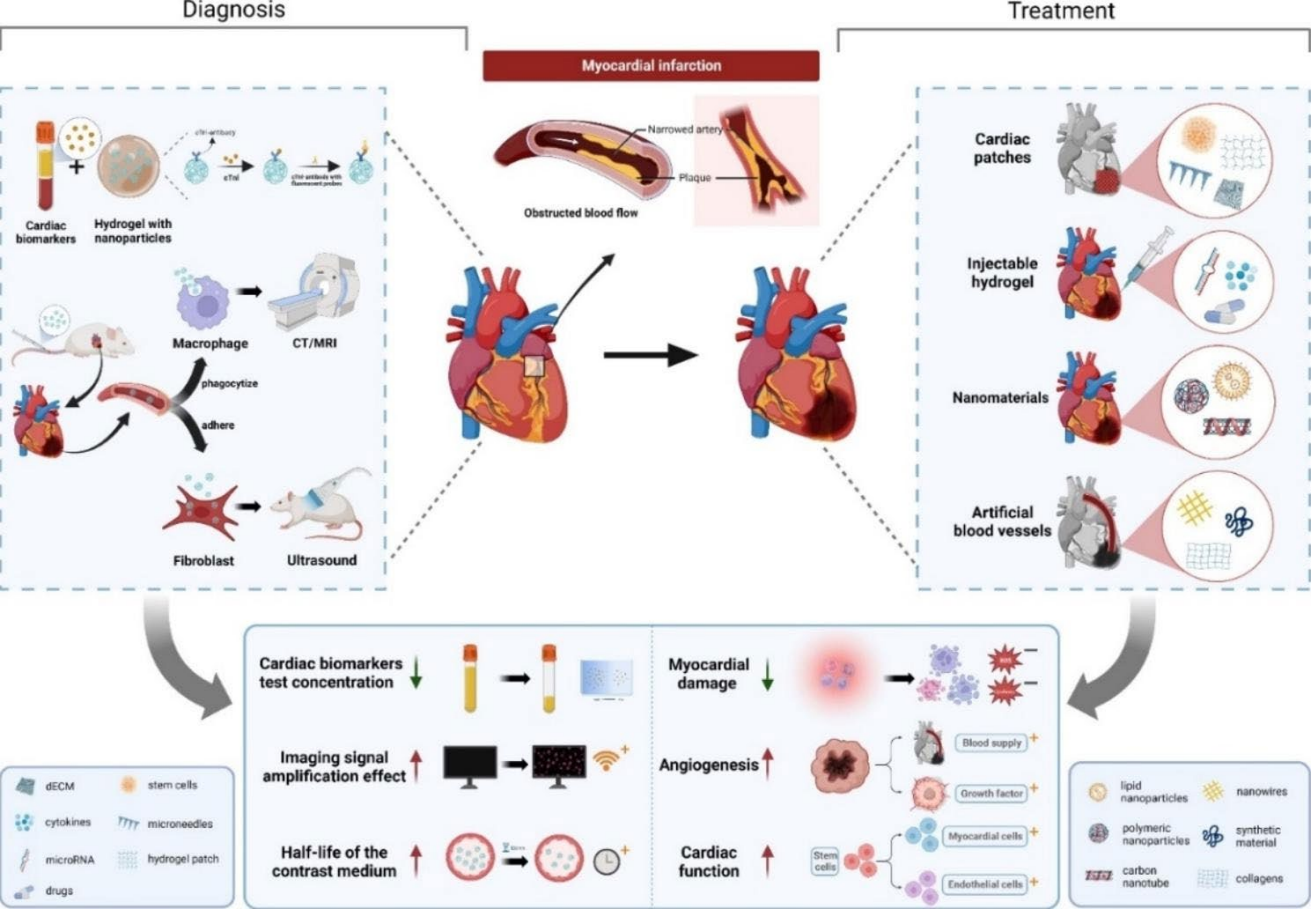
Schematic diagram of the application and effect of biomedical materials in MI diagnosis and treatment

This review summarizes the application progress of biomedical materials in MI diagnosis and treatment. We start from the widely used biomedical materials, such as cardiac patches, hydrogels, nanomaterials, and artificial blood vessels, based on the mechanism of action and therapeutic effect. This review provides a summary of recent relevant studies. We outline the benefits and traits of biomedical materials and discuss the drawbacks discovered through studies. Finally, we summarize these studies with prospects. We present some potential biomedical materials and technologies that have emerged in recent years. (Fig. 1)

## Biomedical materials in MI diagnosis and treatment

Biomedical materials are widely used in the cardiovascular field, especially in MI [31]. Immediately after MI, danger-associated molecular patterns (DAMPs) trigger inflammation by activating pro-inflammatory signaling pathways, such as toll-like receptors and interleukin (IL)-1. These molecules recruit neutrophils and monocytes to migrate to the injured area and clear dead cells [32, 33]. Early inflammatory response leads to myocardial death, ventricular wall thinning, and dilation [34]. After approximately one week, the initial inflammatory response fades away. The fibroblasts in the heart are transdifferentiated into myofibroblasts under the stimulation of growth factor, promoting collagen synthesis and scar tissue formation [35]. This process completes in approximately four weeks, the structural matrix protein network is formed in the infarcted tissue, the role of myofibroblasts is weakened, angiogenesis is inhibited, the scar gradually matures, and ventricular remodeling occurs [36]. Biomedical materials play an irreplaceable role in reducing the inflammatory response in the early stage of MI, restoring reperfusion, and promoting cardiac regeneration in the late stage of MI due to their unique physicochemical properties and cargo-loading capacity. Recent studies have been conducted on cardiac patches, hydrogels, nano biomaterials, and artificial blood vessels [37].

### Cardiac patches in MI diagnosis and treatment

The cardiac patch is in direct contact with the infarcted area, providing a scaffold for the cells in the infarcted area and promoting their healing. Cardiac patches are divided into two parts: the stent and the therapeutic component. Recently, natural or synthetic materials have been used to make cardiac patches. Natural materials include protein- (collagen, fibronectin, and gelatin) [38–40] and polysaccharide-based (chitosan and hyaluronic acid) [40–42]. They have good biocompatibility, degradability, low immune rejection, and no toxicity [43]. Synthetic materials include polyglycerol sebacate (PGS), [44] Polycaprolactone (PCL), [45] polyethylene glycol (PEG), [46] polylactic acid (PLA),[47] polyvinyl alcohol (PVA), and polyvinyl pyrrolidone (PVP) [48]. Synthetic materials have the advantages of easy preparation, good mechanical properties, and low cost [49]. McMahan et al. proposed three requirements for preparing cardiac patches: a physiologically accurate scaffold microstructure, a mechanical structure that supports the dynamics of a beating heart, and proper biocompatibility and degradation rate [50]. This provides the basis for the preparation of cardiac patch scaffolds.

Recently, decellularized extracellular matrix (dECM) has been a research hotspot [51]. It retains the natural tissue structure and blood vessels by removing cellular and nuclear material components from autologous or allogeneic natural tissues [52]. Physical, chemical, and biological methods can prepare it [53]. The cardiac patches prepared have complex biological components, retain the microenvironment of the extracellular matrix, have good biocompatibility, and contribute to the regeneration, repair, and remodeling of damaged myocardium [54, 55]. Microneedles, a new type of biomaterial, has recently been used as scaffolds for cardiac patches and have played a role in MI [56].

### Hydrogels in MI diagnosis and treatment

Hydrogels are polymers with a three-dimensional network structure composed of 90% water. It is widely used in tissue engineering and drug delivery due to its high biocompatibility and molecular permeability [57]. Researchers can modify functional molecules with the desired structure and function, such as conductivity, [58] stimulus responsiveness, [59] and fluorescence effect, [60] through molecular permeability. In MI, the applications of hydrogels mainly include (a) cardiac patches: repairing damaged myocardium through scaffolding, [61] and (b) injectable hydrogels: acting as delivery systems to load biomolecules or drugs [62].

The materials used to synthesize hydrogels must be biocompatible, degradable, non-toxic, and have specific mechanical properties. Both natural and synthetic materials are used for hydrogel synthesis. Natural polymers include alginate, [63] cellulose, [64] chitosan, [65] collagen, [66] and hyaluronic acid [67]. Synthetic polymers include polyethylene glycol (PEG), [68] polyacrylamide (PAM), [69] polylactic acid (PLA), [70] polycaprolactone (PCL), [71] and polyvinyl alcohol (PVA) [72]. MI applications require elasticity and contractility to deform with the heart’s beating. It also needs a certain retention time and controlled release to deliver drugs or biomolecules in vivo [73]. Bar et al. proposed three key criteria for hydrogel design: (a) mechanical stiffness (between 0.1 and 20 kPa); (b) a stable physical and biochemical microenvironment suitable for the survival of loaded substances; and (c) retention time of loading material or itself after implantation [74].

### Nano biomaterials in MI diagnosis and treatment

Nano biomaterials are materials synthesized at the nanometer scale (10^− 9^ m). This review focuses on nano biomaterials for medical applications. In addition to their own physical and chemical properties, they are also capable of acting as nanocarriers. Unlike the invasiveness of the above two biomedical materials, nano biomaterials can achieve targeted therapy and are minimally invasive. Nano biomaterials have different properties than conventional materials due to their high surface area-to-volume ratio. It can interact with target cells or organs at the molecular level. Nano biomaterials can be used as carriers of drugs or bioactive molecules and can be loaded with imaging agents, contributing to the imaging diagnosis of MI [75, 76]. Common forms of nanomaterials include nanotubes, nanofibres, nanorods, nanoparticles (NPs), and thin films [77].

Nanomaterials are made up of various substances, including inorganic and organic molecules. According to recent studies, many nano biomaterials contain gold, silver, silica, iron oxide, and other components. Their unique electromagnetic and optical properties make such biomedical materials have significant advantages in tissue imaging and photothermal therapy [78]. Carbon nanomaterials exhibit high electrical and thermal conductivity, such as carbon nanotubes, graphene, nanodiamonds, and fullerenes [79]. They can interact with proteins through the π − π stacking, van der Waals forces, electrostatic and hydrophobic key [80]. Liposomes are the most common lipid-based nanomaterials. It has good histocompatibility because its surface layer is composed of a phospholipid bilayer. It can carry numerous amphiphilic cargoes and can also bind various functional molecules [81]. Natural or synthetic polymers are used to prepare polymeric nano biomaterials. They have a controlled release effect and can reduce the impact of the microenvironment on the loaded drug, thereby improving drug bioavailability [82]. It is noteworthy that with the application of nanomaterials, more and more of them are released into the environment. In addition to ecotoxicity, we should consider the biological toxicity of nanomaterials, such as cardiovascular and neurotoxicity [83, 84]. Appropriate measures are needed to assess and address nanomaterial toxicity [85].

### Artificial blood vessels in MI diagnosis and treatment

CABG employs autogenous veins, primarily saphenous veins, and internal thoracic arteries [86]. However, autologous blood vessels cannot be removed from MI patients in many cases because their physical condition may not tolerate surgery. Therefore, artificial blood vessels have replaced blood vessel transplantation. The transplanted blood vessels can deliver blood directly to the infarcted area, reducing myocardial cell damage. Artificial blood vessels must not degrade over time and remain unobstructed as a blood channel [87].

Materials used for artificial blood vessels include natural and synthetic materials. Natural materials are biocompatible and can provide an extracellular matrix (ECM), promotes cell adhesion, and contributes to the recovery of infarcted tissue. Common natural materials are collagens, [88] elastin, [89] fibrinogens, [90] chitosan, [91] and cellulose [92]. However, the degradation rate of natural materials is extreme. The emergence of synthetic materials solves this problem. Synthetic materials have controlled degradation rates, better mechanical properties, and are non-toxic. Common synthetic materials are polycaprolactone (PCL), [93] polyethylene terephthalate (PET), [94] polylactic acid (PLA), [95] polycarbonate polyurethane (PCU), [96] thermoplastic polyurethane (TPU), [97] polyglycerol sebacate (PGS), [98] and co-polymers of poly lactic-co-glycolic acid (PLGA) [99]. Artificial blood vessels integrating natural and synthetic materials have been developed. It has the mechanical properties of synthetic materials and good biocompatibility with natural materials [100]. The common methods for synthesizing blood vessels include melt spinning, [101] electrospinning, [102] 3D printing, [103] and gas foaming [104].

## Biomaterial applications in MI diagnosis

Early MI treatment is critical to salvage ischemic myocardium. Therefore, reliable methods are needed for timely MI diagnosis. The MI diagnosis begins with the history and clinical presentation. Electrocardiograms, cardiac biomarkers, and imaging are common diagnostic methods [105]. However, these methods have poor sensitivity and specificity. Additionally, current contrast agents are poorly targeted and rapidly metabolized in vivo. The unique physicochemical and targeting properties of biomedical materials bring hope for rapid MI diagnosis.

### Cardiac biomarkers

Cardiac biomarkers are of great value in early MI diagnosis. Myoglobin (Mb) and heart-type fatty acid-binding protein (h-FABP) are the earliest biomarkers that rise approximately 1–2 h after the onset of MI, but they lack specificity. Cardiac troponins (cTns) began to rise subsequently and are considered the preferred markers for MI diagnosis. Creatine kinase (CK) and creatine kinase isoenzyme (CK-MB) are classic MI biomarkers. Additionally, B-Type natriuretic peptide (BNP), glutathione (GSH), lactate dehydrogenase (LDH), C reactive protein (CRP), and aspartate transaminase (AST) are available, but their specificity and sensitivity are unsatisfactory [106]. Antigen-antibody immunoaffinity detection is used to identify these biomarkers, including enzyme-linked immunosorbent assay (ELISA), electrochemiluminescence (ECL), surface plasmon resonance (SPR), and photoelectrochemical (PEC).

Currently, nanomaterials and hydrogels are primarily used in biomarker detection. Nanomaterials can be utilized in various applications due to their adhesive properties, high permeability, and excellent signal amplification capabilities. Li et al. [107] developed a new dynamic and pseudo-homogeneous ELISA strategy. They used a combination of bioconjugated magnetic nanochains (MNCs) and gold nanoparticles-horseradish peroxidase (AuNPs-HRP) nanoprobes. First, the specimen to be tested was placed together with MNC-Ab1 in a 96-well plate. In the presence of an external magnetic field, the antibody-conjugated MNC acts as a stirrer and trapping agent to bind the tested molecule to Ab1. Then, Ab2-AuNPs-HRP rapidly bound to the target on the surface of MNC to form a sandwich immune complex. Finally, the ELISA substrate was added to produce a blue reaction product, which could be measured by a microplate reader. AuNPs-HRP nanoprobes played a role in signal amplification to achieve highly sensitive detection. This method accurately detected a panel of AMI biomarkers within 35 min. Hydrogels are widely used to detect biomarkers due to their conductivity, hydrophilicity, high stability, and porous structure [108]. The detection time is greatly reduced, and the sensitivity is significantly improved through biomedical materials, ensuring the rescue of ischemic myocardium.

#### Mb

Myoglobin is an iron - and oxygen-binding protein that is abundant in the heart and skeletal muscle of animals. At the time of MI, myocardial cells are damaged and Mb is released into the blood. Therefore, the detection of Mb has some warning effect but no specificity in the early stage of MI [109]. Al Fatease et al. [110] coated chromium-modified zinc oxide nanoparticles (ZnO NPs) onto gold-plated electrodes and used differential pulse voltammetry (DPV) to detect Mb. During cyclic voltammetry (CV), when the applied potential at the electrode crosses the E1/2 value, electrons are transferred from ZnO to Mb, translating it from Fe^3+^ to Fe^2+^ reducing Mb and oxidizing ZnO, resulting in increased oxidation current. During the negative scan, when the applied potential reaches equilibrium, electrons in the Fe^3+^ state to Fe^2+^ cause the current to decrease by releasing electrons into ZnO. Finally, the rapid detection of Mb can be achieved through the characteristic CV curve. Compared with pure zno nanoparticles, the detection sensitivity of Mb is improved by three times. There are also studies using gold nanoparticles decorated boron nitride nanosheets to detect Mb by the DPV method [111]. The covalent interaction of Au-S can immobilize the thiolated DNA aptamer, which can be used to specifically bind Mb. The [Fe(CN)_6_]^3−^/^4−^ was used as a REDOX probe to monitor the oxidation current changes when different concentrations of Mb were bound on the sensor surface. He et al. [112] constructed a dual-probe sensor. One probe is mesoporous carbon nanospheres (MOCs) bound to the luminescent material Ru(bpy)_3_^2+^, whose surface is loaded with Mb aptamers. The other probe consisted of MoS_2_ quantum dots(QDs) loaded with cTnI Ab. Using the ECL method, two different ECL signals will be generated in a single potential scan to simultaneously detect cTnI and Mb. The intensity of ECL reflects the concentrations of cTnI and Mb. Hydrogels can also be fabricated into nanostructures for cardiac biomarkers detection. Singh et al. [113] used nanoengineered mesoporous L-cystein-graphene (Cys-rGO) hydrogel combined with microfluidics to detect Mb by SPR and DPV. In DPV mode, the generated current was measured before each potential change, and the current difference was plotted against the potential. For SPR measurements, the specific Mb Ab on the electrode surface was first fixed. When the target molecule flows on the Au surface, its binding is facilitated through affinity interactions and subsequently the sensor surface refractive index enhancement is induced. The mesoporous structure, high surface area, and electrical conductivity of hydrogels are favorable for detection. Although Mb is the earliest elevated biomarker in MI, a diagnosis is often uncertain due to its low specificity.

#### CK-MB

CK is an enzyme that catalyzes the reversion of creatine and ATP to phosphocreatine and ADP. CK is present in the mitochondria and cytoplasm of muscle cells, and two subunits, M and B, form a dimeric enzyme, specifically, in three forms: CK-BB, CK-MB, and CK-MM. CK-MM was expressed in all tissues. CK-BB is mainly found in the brain, kidney, and gastrointestinal tract. CK-MB is present in the heart, skeletal muscle, uterus, small intestine, etc. Approximately 20% of CK in the myocardium is in the MB form, which increases sensitivity and specificity for the diagnosis of MI [114, 115]. CK-MB appears earlier (within 4 h) in the blood of MI patients. CK-MB has a higher specificity than Mb and is a diagnostic marker for early MI. Chen et al. [69] designed a DNA hydrogel microfluidic chip-mediated point-of-care test (POCT) platform to detect CK-MB. In the presence of CK-MB, the cross-linking density was reduced by competing for target aptamer binding, thereby collapsing the hydrogel and releasing the pre-encapsulated gold nanoparticles for colorimetric detection. The limit of detection (LOD) was as low as 0.027 nM. A portable, low-cost, high-detection rate and reusable CK-MB detection platform have been established through the sensitivity of hydrogel and the signal amplification of nanoparticles. Lai et al. [116] used fluorescence immunochromatographic assays (FL-ICAs) combined with Eu (III) chelate polystyrene microparticles (CM-EUs) has designed a rapid and simple method to detect CK-MB, which is a sandwich immunoassay. CK-MB-Ag in the sample binds to (CM-EU) -Ab1. After reaching the detection line, it binds to Ab2. CM-EU-RIgG then further migrated to the control line and reacted with anti-rigG. Finally, the test strip was analyzed by measuring the height of the fluorescence peak of the test and control lines using a TRF reader. The detection range was 0.85–100.29 ng mL^− 1^, and the LOD was 0.029ng mL^− 1^. In addition, the high surface area of 3D gold nanocapsules enables the detection of a variety of cardiac markers by the EFISA method. The excellent stability, conductivity and signal amplification effect of carbon nanomaterials have also been used for CK-MB detection by ECL method [117, 118].

#### cTns

Troponins in the myocardium form heterotrimers: consisting of troponin I (TnI), T (TnT), and C (TnC) as subunits. They interact with tropomyosin as part of cardiac sarcomere filaments, regulating the calcium-dependent interaction of actin and myosin in response to changes in cytosolic calcium. TnC is also present in striated muscle and is not suitable for the diagnosis of MI. Circulating troponin levels in healthy individuals are very low, so troponin plasma levels can easily identify small myocyte damage. Cardiac myocytes possess a very small cytosolic troponin pool, and the majority of troponin is located within the contractile apparatus of these cells. After MI, troponin is released initially from the cytoplasmic pool and later from the contractile apparatus of damaged cells [119]. Cai et al. [120] employed a polystyrene core and a polyacrylic shell to compose nanoparticles. The former embedded the fluorescent dye Nile red, and the latter loaded Ab1 for binding to cTnI. A new POCT platform was established. Samples were added to the sample pad and migrated toward the cTnI McAb1 particles, and the complex was then captured with cTnI McAb2, which was coated on the NC membrane as a T-line. Next, the C-line captures the excess pellets. Finally, the excess nanoparticles migrate into the absorption pad by capillary action. After the immune reaction process, the fluorescence intensity was estimated by fluorescent microspheres on T-and-C-lines. The minimum detection concentration could be as low as 0.016 ng ml^− 1^, and the detection time was only 15 min. Polyethylenimine-functionalized graphene nanocomposite, Ag2S/ZnO nanocomposites, hexagonal boron nitride nanosheets, and gold nanomaterials were also utilized for cTnI detection due to their strong signal amplification, electrical conductivity, and high surface area [121–125]. The nano biomaterials load more primary antibodies through their high surface area, increasing cTns uptake. Moreover, the electron transport properties of metal nanomaterials play a signal amplification effect and enhance the observable signal of cTns. Additionally, hydrogel materials are applied to detect cTnI. Ji et al. [126] designed a porous hydrogel-encapsulated photonic crystal (PhC). cTnI, BNP, and Mb were detected by EFISA method in suspension array. Ab1 encapsulated on PhC can bind to Ag in the sample, followed by incubation with Cy3-Ab2 to produce a fluorescent effect to detect a variety of cardiac markers. The LODs were 0.009 ng mL^− 1^, 0.084 pg mL^− 1^, and 0.68 ng mL^− 1^, respectively. Molecules could diffuse across the interconnected nanochannels due to the hydrophilic nature of the hydrogel, making the test stable.

#### Other biomarkers

GSH, h-FABP, and BNP are also elevated at MI but lack diagnostic specificity. GSH is an important tripeptide thiol that acts as an antioxidant, and its intracellular concentration is an indicator of oxidative stress [127]. MI can trigger the production of free radicals. The massive production of free radicals after myocardial ischemia usually leads to the depletion of GSH. Carbon dots (CDs) were used for detecting GSH by the PEC method due to their optoelectronic and catalytic properties. Reducing agents such as GSH can be catalyzed by photoexcited holes in the CDs core to oxidize to GSSG, resulting in positive photocurrent. Larger concentrations of glutathione can accelerate this process, resulting in larger photocurrent amplitudes. Therefore, the GSH concentration can be determined by the enhancement of the positive photocurrent amplitude at an applied voltage greater than − 100 mV. CDs enable this sensing process to be further enhanced by binding to silver nanoparticles (AgNPs), graphene oxide (GO), and mesoporous silica (MS). The LOD of GSH was 6.2 nM [128].

FABP is a non-enzymatic protein involved in intracellular buffering and transport of long fatty acid chains, protecting cardiomyocytes from long-chain fatty acids. h-FABP is one of nine specific FABP families [129]. Similar to Mb, h-FABP is a protein present in cardiomyocytes. It is of great value for the diagnosis of MI. Li et al. [130] designed two CS hydrogel beads, in which cTnI and h-FABP antibodies were bound by chitosan and AuNPs on the surface. When the samples were added, antibodies to the two hydrogel beads could capture their antigens separately. When H2O2 was added, the fluorescein in the two hydrogel beads appeared blue and green when reacting with H2O2, respectively, showing a strong and uniform signal in chemiluminescence (CL). In this way, the linear range for detection of cTnI and h-FABP ranged from 1.0 × 10^–11^ to 1.0 × 10 ^–5^ g mL^− 1^, with LODs as low as 1.57 and 1.61 pg mL^− 1^, respectively. Hydrogel beads have high biocompatibility, monodispersity, and stability.

The ventricular cells secrete BNP. After synthesis, the proBNP precursor is cleaved into active BNP and inactive N-terminal pro-B-type natriuretic peptide (NT-proBNP). BNP/ NT-proBNP has a certain predictive effect on heart failure, which is significant in evaluating the degree of cardiac damage and prognosis after MI [131]. Dong et al. [132] used semicarbazide-modified gold nanoparticles (AgNC-sem@AuNPs) to cover silver nanocubes, NT-proBNP was detected in ECL assay by the formation of sandwich immune complexes. Notably, Ab2 binding leads to the quenching of ECL. The ECL intensity decreased with increasing NT-proBNP concentration. The lower limit of detection was 0.11 pg mL^− 1^. Liu et al. [133] used In2O3 nanoribbon biosensors to detect cTnI, BNP, and CK-MB by electronic enzyme-linked immunosorbent assay. After the antigen-antibody sandwich immune complex is formed, biotinylated urease is introduced. It reacts and consumes protons, which raises the pH of the solution. Further, it leads to deprotonation of hydroxyl groups on the surface of In2O3 nanoribbon, which ultimately reduces the surface potential. The catalytic reaction facilitated by urease amplifies the charge generated by analyte binding by several orders of magnitude, which makes the pH change easily detected by the In2O3 nanoribbons sensor. From this potential change, the concentration of the biomarker can be detected. Biomarkers were detected at concentrations as low as 1 pg mL^− 1^ (cTnI), 0.1 ng mL^− 1^ (CK-MB), and 10 pg mL^− 1^ (BNP) over a 45-min period.

The experiments mentioned above demonstrate that the high surface area of biomedical materials can improve the embedding capacity of primary and secondary antibodies. Electron transport properties and enzyme-like materials can exert signal amplification effects. Biomedical materials offer low cost, high efficiency, sensitivity, and specificity to detect cardiac biomarkers. The development of new detection techniques has significantly reduced the detection time. The combination of biomedical materials and POCT platforms has facilitated detection. The advantages and limitations of representative studies that detect cardiac markers are summarized. (Table 1) Cardiac biomarker detection is a promising application for biomedical materials.

**Table 1:** Representative studies of biomedical materials in cardiac biomarkers detection

Biomedical materials	Biomarkers	Method	Advantages	Limitations	Ref
AuNPs\ MNC	cTnI, Mb, CK-MB, CRP	ELISA	Fast and high sensitivity, high selectivity and reliability.	Requires a specific magnetic field environment.	[107]
ZnO NPs\Cr	Mb	DPV	Fast and high sensitivity.	Low detection efficiency, single detection type.	[110]
AuNP/BNNS	Mb	DPV	High signal response for Mb.	Sensitivity is not satisfactory, single detection type.	[111]
MOCs\ QDs	cTnI, Mb	ECL	High sensitivity, excellent specificity, selectivity and stability.	Few detection types.	[112]
Cys-RGO	Mb	SPR\ DPV	Low LOD, high accuracy, high-sensitivity and high binding kinetics.	The sensitivity of the two methods is different, single detection type.	[113]
AuNPs\ DNA hydrogel	CK-MB	Colorimetric detection	Good portability, visualization, and simple sample handling.	The method of obtaining results is not simple.	[69]
CM-EUs	CK-MB	FL-ICA	Simple, inexpensive system that provides quantitative, sensitivity and reliable detection.	Long detection time.	[116]
GNV	NT-proBNP, CK-MB, cTnT	EFISA	Wide linear range, high precision.	Complex detection mode.	[117]
CNOs/AuNPs/Fe_3_O_4_/Chi	CK-MB	ECL	Better sensitivity, without the use of harmful-complex components, enzymes, time-consuming pre-treatments.	Single detection type.	[118]
Polystyrene\ polyacrylic acid	cTnI	LFIA	A rapid, accurate, and stable assay.	Single detection type.	[120]
PEI\ GO	cTnI	ECL	High sensitivity, good selectivity and good accuracy for detection.	Single detection type.	[121]
Ag_2_S/ZnO\ Au NPs	cTnI	PEC\ CRCA	High sensitivity and good stability.	Single detection type, Average accuracy.	[122]
BNQD	cTnI	DPV	High stability, repeatability, reproducibility and reusability, high selectivity and sensitivity.	Single detection type.	[123]
AuNPs	cTnI	ECL	High sensitivity, high specificity.	Single detection type, long detection time.	[124]
GNR	cTnI, MG	Label free protein detection	Quick, reliable, high sensitivity.	Few detection types, long detection time.	[125]
PhC	cTnI, BNP, Mb	EFISA	Highly sensitive, flexible, and high-throughput.	Long detection time.	[126]
CD\ AgNPs\ GO\MS	GSH	PEC	Good selectivity, stability and reproducibility.	Reliability of the fabrication processes still needs to be improved.	[128]
CS hydrogel beads\ AuNPs	cTnI\ h-FABP	CL	Wide linear range, low detection limit, good selectivity, high stability and good operability.	Few detection types.	[130]
AgNC-sem@AuNPs	NT-proBNP	ECL	Favorable sensitivity, selectivity and repeatability.	Complex detection mode, single detection type.	[132]
In_2_O_3_ nanoribbon	cTnI, BNP, CK-MB	Electronic ELISA	High sensitivity, quick turnaround time, and reusability.	Other possible materials may also be suitable to work as channel materials, which need further investigation.	[133]

### Imaging

Magnetic resonance imaging (MRI), computed tomography (CT), and ultrasound (US) are common imaging diagnostic techniques that distinguish infarcted myocardium from healthy myocardium. However, imaging has limitations in MI diagnosis due to the rapid metabolism and lack of contrast agent targeting. New contrast agents are urgently required to make up for the current shortfall. Biomedical materials can achieve targeting, controllable metabolic rates, and signal amplification. The advantages and limitations of representative studies of imaging tests for MI are summarized. (Table 2)

**Table 2:** Representative studies of biomedical materials in imaging tests for MI

Biomedical materials	Imaging modalities	Advantages	Limitations	Ref
DSPE-PEG\ MFRFs	MRI	Qualitative and quantitative MRI, high accumulation.	High cost.	[135]
Hsp70-SPIONs		High contrast, preferential accumulation in the heart tissue.	Low sensitivity.	[136]
MnO_2_@BSA		High spatial resolution, good molecular relaxivity, high accumulation, rapid metabolism.	Long scan times.	[137]
AuNPs	CT	High tissue accumulation, prolonged and significant imaging, adequate contrast, superior tissue penetration and spatial resolution, high safety.	Radiation, poor soft tissue contrast.	[138]
AuNPs		Stable blood pool enhancement, superior X-ray attenuation and profile.	Radiation, amount of AuNPs necessary for imaging was relatively high, lower limb dysfunction of unknown cause.	[139]
*c*ROMP nanoparticles		High tissue accumulation, good structural integrity, high sensitivity.	Radiation, uncertain eliminating method.	[140]
N1177		Good targeting, high spatial and temporal resolutions.	Radiation, uncertain kinetics and optimal dose.	[141]
ExiTron MyoC 8000		High iodine concentration, long blood half-life, ingested by healthy heart muscle, low dose required.	Radiation, poor targeting.	[142]
CNA35-PFP NPs	US	High contrast, high tissue accumulation, noninvasive, economical, and real-time.	Low resolution.	[143]
CLIO-VT750	FMT	Long half-life, high contrast, good targeting, multi-channel simultaneous imaging.	Long scan times.	[144]
Macroflor	PET	Short blood half-life, high tissue accumulation, good targeting.	Lower retention, long scan times.	[145]
^64^Cu-Macrin		Quantitative determination, good targeting, high safety.	Imaging depends on the abundance, activity, and phagocytic history of macrophages.	[146]

#### MRI

Cardiac MRI has a high temporal and spatial resolution, as well as excellent soft tissue contrast. Therefore, it can exhibit different tissue changes in the infarct area after MI, such as hemorrhage, edema, microvascular obstruction and fibrosis. The extent and degree of these pathological changes are important for MI diagnosis and prognosis. Therefore, cardiac MRI is also considered the gold standard for MI non-invasive diagnosis [134].

**Fig. 2:**
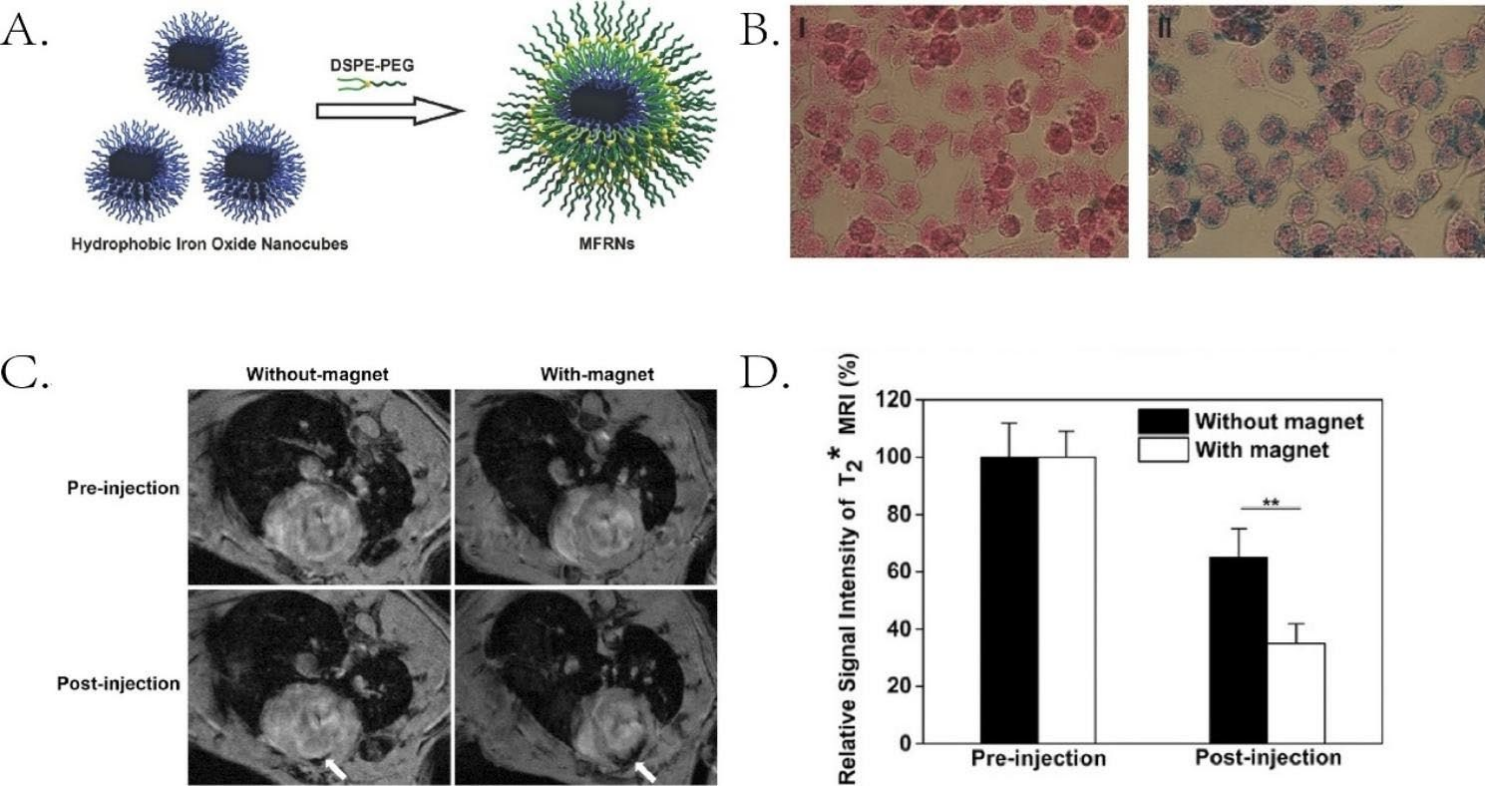
DSPE-PEG encapsulated hydrophobic iron oxide nanocubes for targeted MRI detection. (**A**) Schematic illustration of improving the colloidal stability and biocompatibility of hydrophobic nanocubes using DSPE-PEG. (**B**) Prussian blue staining of RAW264.7 cells cultured without (I) or with (II) MFRN. Cells were stained in red and MFRN in the cytoplasm was stained in blue. (**C**) In vivo 7 Tesla cardiac MR images before and 24 h after intravenous injection of MFRN, and with or without external magnetic exposure measures. White arrows point to infarcted tissue. (**D**) Negative contrast is stronger with injection of MFRN and with magnetic exposure. Reproduced with permission [135]. Copyright 2016, Wiley-VCH

Nanomaterials can aggregate in the heart by targeting cells in the MI infarct zone. Macrophages can phagocytose iron oxide nanoparticles. Hu et al. [135] encapsulated hydrophobic iron oxide nanocubes in the hydrophilic material 1,2-dioleoyl-sn-glycero-3-phosphoethanolaminen- [poly (ethylene glycol)] (DSPE-PEG). Iron oxide nanocubes are Magnetic field responsive nanocubes (MFRFs) with magnetic targeting properties under the action of an external magnetic field, enabling qualitative and quantitative imaging of cardiac tissue by MRI. Macrophage phagocytosis facilitated tissue infiltration. (Fig. 2) The stability, magnetic properties, and phagocytosis of iron oxide nanomaterials by macrophages enabled the qualitative and quantitative detection of MI using MRI. The Binding of different ligands can enhance the properties of iron oxide nanoparticles further. Shevtsov et al. [136] combined 70-kDa heat shock protein (Hsp70) with superparamagnetic iron oxide nanoparticles (SPIONs) as Hsp70- SPIONs and discovered that it could penetrate the infarcted area via CD40-mediated endocytosis. Hsp70-SPIONs may accumulate faster than Pure SPIONs in cardiac tissue, allowing MRI to detect MI faster and more accurately. Furthermore, a pH-responsive MnO_2_ nanocomplex has been designed to target inflammatory cells in MI [137]. Mn^2+^ from MnO_2_ is released in an acidic environment. The interaction between Mn^2+^ and environmental albumin increases relaxation. Nanomaterials are injectable, magnetic, and targetable materials crucial to MRI imaging.

#### CT

CT is widely used to diagnose MI due to its rapidity, high resolution, and non-invasiveness. The use of biological materials improves the accuracy of CT detection. The unit weight contrast of AuNPs is three times that of iodinated contrast agents. Danila et al. [138] used AuNPs encapsulated by the collagen-homing peptide (CNA35), which could target myocardial scars and exert an enhanced effect on MI scars in mice, enabling prolonged and dramatic imaging. The high surface area of ​​AuNPs could load more CNA35. AuNPs have better tissue retention relative to iodinated contrast agents, thus increasing their concentration in the myocardium. Furthermore, AuNPs can provide stable blood pool enhancement in rats [139]. Pan et al. [140] also designed nanomaterials with similar functions. Soft colloidal, radio-opaque, and metal-encapsulated polymeric (cROMP) Nanoparticles allowed for aggregation to specific targets in blood vessels. Furthermore, cROMP nanoparticles did not extravasate into tissues due to their stability and integrity. The half-life of cROMP nanoparticles for CT imaging had six times longer than iodinated contrast agents. These studies provide new ideas for CT angiography. It has potential application value in MI detection and diagnosis. Hyafil et al. [141] used an iodinated nanoparticle contrast agent N1177 that macrophages could take up to detect macrophage infiltration in atherosclerotic plaques. This is significant for the early MI diagnosis caused by plaque rupture. Sawall et al. [142] developed a novel blood pooling agent, ExiTron MyoC 8000. It had a high iodine concentration (210 mg mL^− 1^), a long blood half-life (~ 2 h), and healthy myocardium could absorb it. Therefore, MI could be displayed even with low-dose injections. This method enabled the quantification of infarct size.

#### Other imaging methods

Ultrasound is a convenient, fast, low-cost, and non-invasive imaging modality. Zhou et al. [143] designed multifunctional CNA35-labeled perfluoropentane nanoparticles (CNA35-PFP NPs). In the MI rabbit model, CNA35-PFP NPs could effectively adhere to the surface of fibroblasts in the fibrotic myocardium. The temperature-sensitive PFP material could be transformed into a gaseous state under ultrasonic irradiation. It significantly improved ultrasound contrast in fibrotic areas. Fluorescence molecular tomography (FMT) can also be used for MI imaging. CLIO-VT750 is a magnetic fluorescent iron oxide nanoparticle designed for FMT imaging of inflammatory cells in MI [144]. Macrophages and neutrophils could phagocytose CLIO-VT750. Therefore, myocardial injury and repair in MI-infarcted areas could be detected using FMT imaging. Additionally, positron emission tomography (PET) imaging can detect inflammation in the infarcted area. Keliher et al. [145] designed Macroflor, a polydextrose nanoparticle with a high affinity for macrophages. Nahrendorf et al. [146] used nanotracer ^64^Cu-Macrin and also reached the same conclusion. The macrophage accumulation in the MI infarct area can be quantified using PET imaging.

## Biomaterial applications in MI therapy

Existing treatments focus on restoring the reperfusion of occluded vessels. However, the infarcted myocardium cannot be rejuvenated even when blood flow is restored. Therefore, severe MI patients eventually develop heart failure. Furthermore, the inflammatory response is crucial in the early MI stage. Effective methods to suppress inflammation and immune responses are worth exploring. Biomedical materials demonstrate novel therapeutic strategies in MI to reduce myocardial injury, promote angiogenesis and improve cardiac function. The advantages and limitations of representative studies of biomedical materials in MI treatment are summarized. (Table 3)

**Table 3:** Representative studies of biomedical materials in MI treatment

Biomedical materials	Model & Method	Function	Advantages	Limitations	Ref
TAK-242- PLGA- NP	Mouse, i.v.	Inhibits TLR4 and inflammatory cell aggregation.	Specificity and targeting, non-invasive, good persistence and effect.	The infarct size cannot be reduced, undesired accumulation.	[148]
SPIONs	Cell culture	Inhibits TLR4 and inflammatory cell aggregation.	Excellent biocompatibility, can be swallowed by cells.	Cause an inflammatory response.	[149]
Gel@MSN \ miR-21-5p	Pig, i.m.	pH-responsive hydrogel, inflammation suppression and angiogenesis enhancement.	Injectable, effective site-specific drug delivery, decrease in systemic side effects.	Drug release impacted by pH changes, uncertain electrical coupling with the host.	[150]
MNPs\Alg	Rat, i.m.	Antioxidation and macrophage polarization.	Excellent biocompatibility, injectable, effective site-specific drug delivery, good cell retention.	Non-responsive, drug release depends on natural degradation of hydrogels.	[63]
eNABs\HAL\ MSN	Rat, i.v.	Produces bilirubin, which acts as an anti-inflammatory and reprogramming macrophage.	Non-invasive, good persistence and effect, high tissue aggregation.	Difficult to achieve local application.	[151]
PFTU/Gt	Rat, cardiac patch implantation.	ROS-responsive. ROS levels, lipid peroxidation and expression of related genes were decreased.	Good cell retention and mechanical support, no obvious cytotoxicity.	Requires surgery, electrical coupling through large areas.	[154]
NIPAAm-PEG1500	Sheep, i.m.	Temperature-responsive, reduce ROS levels.	Effective site-specific drug delivery, decrease in systemic side effects.	Inability to precisely determine spatial distribution of hydrogel in vivo, foreign body reaction and fibrosis.	[155]
CSCl-GSH	Cell culture	Temperature-responsive, reduce ROS levels.	Excellent biocompatibility, good cell retention.	The intrinsic pathway should be examined in the future study.	[156]
RGD/PEG-PUE-SLN	Rat, i.v.	Anti-oxidative stress, promote angiogenesis.	Excellent entrapment efficiency and drug loading capacity, excellent biocompatibility, non-invasive.	Long half-life may cause undesired accumulation.	[157]
UCCy@Gel	Mouse, i.m.	Light energy conversion by UCNPs was used for photosynthesis in cyanobacteria to achieve appropriate oxygen liberation.	Both preventive and therapeutic functions, excellent biocompatibility.	Invasive modality, long-term effects in large animals remain to be verified, uncertain metabolism and long-term biosafety.	[159]
GC\PNIPAM	Mouse, i.m.	Temperature-responsive, delivery of VEGF\ IL-10\PDGF.	Quick delivery, high loading efficiency, minimal burst release, sustained release.	Uncertain drug interactions and delivery sequence.	[161]
PVL-b-PEG-b-PVL HG	Rat, i.m.	Temperature-responsive, delivery of VEGF.	Sustained local release, high loading efficiency, improve protein stability, solubility, and biocompatibility.	Invasive modality.	[162]
NFs	Pig, i.m.	Delivery of VEGF.	Precise direct serial delivery, high loading efficiency, high safety, slow degradation.	Invasive modality.	[163]
*p*[NIPAAm-co-PAA-co-BA]	Rat, i.m.	Temperature-and pH-responsive, delivery of bFGF.	Sustained release and local delivery, high loading efficiency, provide spatial and temporal control.	Invasive modality, cause an inflammatory response.	[164]
CS	Rat, i.m.	Delivery of bFGF.	High loading efficiency, controlled mechanical properties and good swelling stability.	Invasive modality, non-responsive, drug release depends on natural degradation of hydrogels.	[165]
NFs	Mouse, i.p.	Delivery of pro-HGF.	High loading efficiency, long half-life, non-invasive.	Undesired accumulation.	[166]
P(CS–CA–NIPAM)	Rat, i.m.	Temperature-and pH-responsive, delivery of OSM.	Continuous and localized release, high loading efficiency, good mechanical support.	Invasive modality.	[168]
BG-SA	Rat, i.m.	Delivery of BG.	Better cardiac retention, avoiding tissue exposure, high loading efficiency, sustained release and local delivery.	Invasive modality, anti-inflammation and anti-oxidative stress should be investigated in a future study.	[170]
RBM-MSC/H-HG	Rat, i.m.	Delivery of heparin and BM-MSC.	Increase cells retention and engraftment, excellent biocompatibility, injectable.	Invasive modality, long-term effects in large animals remain to be verified.	[171]
MN-CSC	Pig, rat, cardiac patch implantation.	Delivery of CSC.	Excellent biocompatibility, high delivery efficiency, good mechanical support.	Requires surgery, uncertain side effects of degradation products.	[172]
NO-RIG	Mouse, i.m.	Temperature- responsive, produce NO in vivo, reduce ROS levels.	NO sustained release and redox equilibrium, injectable, high conductivity.	Invasive modality, Undesired accumulation.	[175]
Col-CNF	Rat, scaffold graft.	Physicochemical property.	High surface area, high conductivity, good mechanical support.	Requires surgery.	[176]
NanoGraft	Pig, artificial blood vessel graft.	Physicochemical property.	The mechanical properties and surface area of the grafts are enhanced.	Occlusion, platelet adhesion.	[178]
PHEA-PLA / PCL	Pig, artificial blood vessel graft.	Physicochemical property.	Good biocompatibility and physicochemical properties.	Thrombosis.	[95]
OGGP3	Rat, i.m.	Physicochemical property.	High conductivity, excellent biocompatibility.	Invasive modality, ability to load drugs should be investigated.	[181]
POG	Rat, cardiac patch implantation.	Physicochemical property.	High conductivity, elasticity, compressive resistance and good biocompatibility, rapid self-healing property.	Requires surgery, ability to load drugs should be investigated.	[182]
EGC scaffolds	Rat, pig, i.m.	Physicochemical property.	High mechanical properties, shape memory and high electrical conductivity.	Invasive modality.	[183]
HA	Rat, i.m.	Delivery of hESC-CMs.	High mechanical properties, increase cells retention and engraftment, excellent biocompatibility, high delivery efficiency.	Invasive modality, requires large cell quantities.	[185]
HHA@ODS	Rat, i.m.	Delivery of MSCs.	Strong adhesion, high delivery efficiency, long retention time.	Invasive modality, further improve the wet adhesive strength.	[186]
Collagen scaffold	Pig, i.m.	Delivery of MSCs.	Promoting cell retention, excellent biocompatibility, injectable, high delivery efficiency.	Invasive modality.	[187]
PFC-conjugated hydrogels	Cell culture	Delivery of MSCs, temperature- responsive, increase oxygen partial pressure,	Excellent biocompatibility, soft and flexible, improve cell survival.	In vivo studies are needed.	[188]
PEG -based hydrogel	Rat, i.m.	Delivery of iPSC-CM.	Excellent biocompatibility, high mechanical properties, injectable.	No engrafted cells were observed at the injection site.	[189]
ECM hydrogel	Mouse, i.m.	Delivery of 7AP.	Excellent biocompatibility, high permeability, good cell retention.	Need long-term evaluation,	[62]
ECM hydrogel	Rat, pig, i.m.	Promoting CSC aggregation	Excellent biocompatibility, high mechanical properties, excellent clinical transformation value.	Long-term effects in large animals remain to be verified.	[191]
BP-NCD	Mouse, epicardium injection.	Delivery of miR.	Good chemical stability, high water solubility, low toxicity, multifunctional surface functions.	Invasive modality.	[192]
miNPs\ shear-thinning hydrogel	Rat, i.m.	Delivery of miR.	Extremely high uptake efficiency, good retention, high biocompatibility.	Invasive modality, requires high dosages.	[193]

### Reduce myocardial injury

An inflammatory response is triggered immediately in the early stage of MI. Inflammatory mediators and proteases are released as inflammatory cells are catalyzed into the infarcted area. This accelerates cardiomyocyte death. Biomedical materials can reduce myocardial injury by inhibiting inflammatory responses, modulating immunity, and removing inflammatory mediators.

M1 macrophages play a proinflammatory role during MI. It activates high mobility group protein B1 (HMGB1), a chromosomal protein in the nucleus that promotes transcription, replication, and DNA repair. It is converted into pro-inflammatory factors in the cytoplasm. HMGB1 interacts with receptors for advanced glycation end products (RAGE) and toll-like receptor (TLR)2/TLR4. The signal transduction occurs through nuclear factor kappa B (NF-κB) and extracellular signal-regulated kinases (ERK)1/2. The pro-inflammatory factors and cytokines expressions, such as IFNγ, IL-6, IL-1β, TNFα, TNFR1, and COX2, are up-regulated [147]. Fujiwara et al. [148] designed poly-(lactic-co-glycolic acid) nanoparticles containing TAK-242 (TAK-242-PLGA-NP). TAK-242 is a TRL4 inhibitor administered intravenously during ischemia-reperfusion (IR). The nanoparticles could inhibit inflammatory cell aggregation. HMGB1 expression in the circulation was reduced, cardiac NF-κB activation and cytokine expression were also decreased, and ventricular remodeling was alleviated. SPIONs also exerted a similar TLR4 inhibitory effect [149]. Li et al. [150] designed pH-responsive hydrogels loaded with microRNA-21-5p (miR-21-5p) silica nanoparticles (MSN). MSN can inhibit M1 macrophages, and miR-21-5p has an angiogenesis-promoting effect. The pH-responsive hydrogels can release MSNs in acidic environments. This composite’s synergistic anti-inflammatory and pro-angiogenic effects reduced infarct size in the porcine MI model.

In addition to inhibiting M1 macrophages, the anti-inflammatory effect can suppress inflammation by promoting the transformation of macrophages into M2 macrophages. Zhou et al. [63] constructed melanin nanoparticles (MNPs)/alginate (Alg) hydrogels through Ca^2+^ crosslinking. The hydrogel encouraged M1 macrophages to convert into M2 macrophages, decreasing CD86 signaling and increasing CD206 signaling in mononuclear macrophages. The hydrogel could scavenge reactive oxygen species (ROS) in a rat model of MI. The oxygen produced by reacting with ROS could promote the polarization of macrophages into the M2 phenotype. The nanoparticles’ specific components can function independently of any additional substances. They can be carried in injectable hydrogels. The hydrogel can provide mechanical support for the sustained and stable release of nanoparticles in the MI infarct area. After neutrophils play an early pro-inflammatory role, macrophages phagocytose apoptotic cells. This leads to the resolution of inflammation and initiation of M2 macrophage transformation. Bao et al. [151] constructed natural neutrophil apoptotic body membranes and engineered neutrophil apoptotic bodies (eNABs) made of mesoporous silica nanoparticles. Hexyl 5-aminolevulinate hydrochloride (HAL) was loaded into it, producing bilirubin intracellularly and exerting anti-inflammatory and reprogramming effects on macrophages. After intravenous injection of nanoparticles in a rat MI model, they promoted M2 macrophage transformation and attenuated ventricular remodeling.

ROS is generated in the ischemic myocardium. ROS leads to Ca^2+^ imbalance in the myocardium and cell death. ROS/JNK, ASK-1/JNK, and other signaling pathways promote cardiomyocyte apoptosis [152]. Inflammatory cells and MMPs can be triggered by ROS as well, accelerating the remodeling of harmed myocardium [153]. Xie et al. [154] synthesized a composite polyurethane (PFTU)/gelatin (PFTU/Gt) ROS-responsive cardiac patch. In the rat MI model, this patch could reduce ROS levels, lipid excess oxidation, and related gene expression, thereby alleviating oxidative stress, inhibiting cell apoptosis, and reducing the inflammatory response. Stimuli-responsive hydrogels function in response to specific stimuli. Spaulding et al. [155] used PEG and N-isopropyl acrylamide (NIPAAm) to synthesize thermoresponsive hydrogels. The thermoresponsive hydrogel gelled at 35 ℃. ROS cleaved PEG, allowing it to be scavenged. In the sheep MI model, the hydrogel was injected into the myocardium in the MI marginal zone. The hydrogel successfully reduced ROS and improved the contractility of the myocardium in the marginal zone. Li et al. [156] also used thermoresponsive chitosan chloride-glutathione (CSCl-GSH) hydrogels. Effectively scavenged ROS in MI to alleviate oxidative stress damage. Puerarin (PUE) is a widely used antioxidant in CVD. Cyclic arginyl-glycyl-aspartic acid (RGD) peptide can target the highly expressed α_v_β_3_ integrin on endothelial cells during angiogenesis in ischemic tissue. Dong et al. [157] developed RGD-modified and PEGylated solid lipid nanoparticles loaded with puerarin (RGD/PEG-PUE-SLN). The nanoparticles significantly increased the circulation time of PUE in serum. The intravenous injection into the serum of the MI rat model significantly reduces myocardial infarct size. The nanoparticle application targeted the PUE delivery to the ischemic myocardium and significantly increased PUE in blood circulation time.

**Fig. 3:**
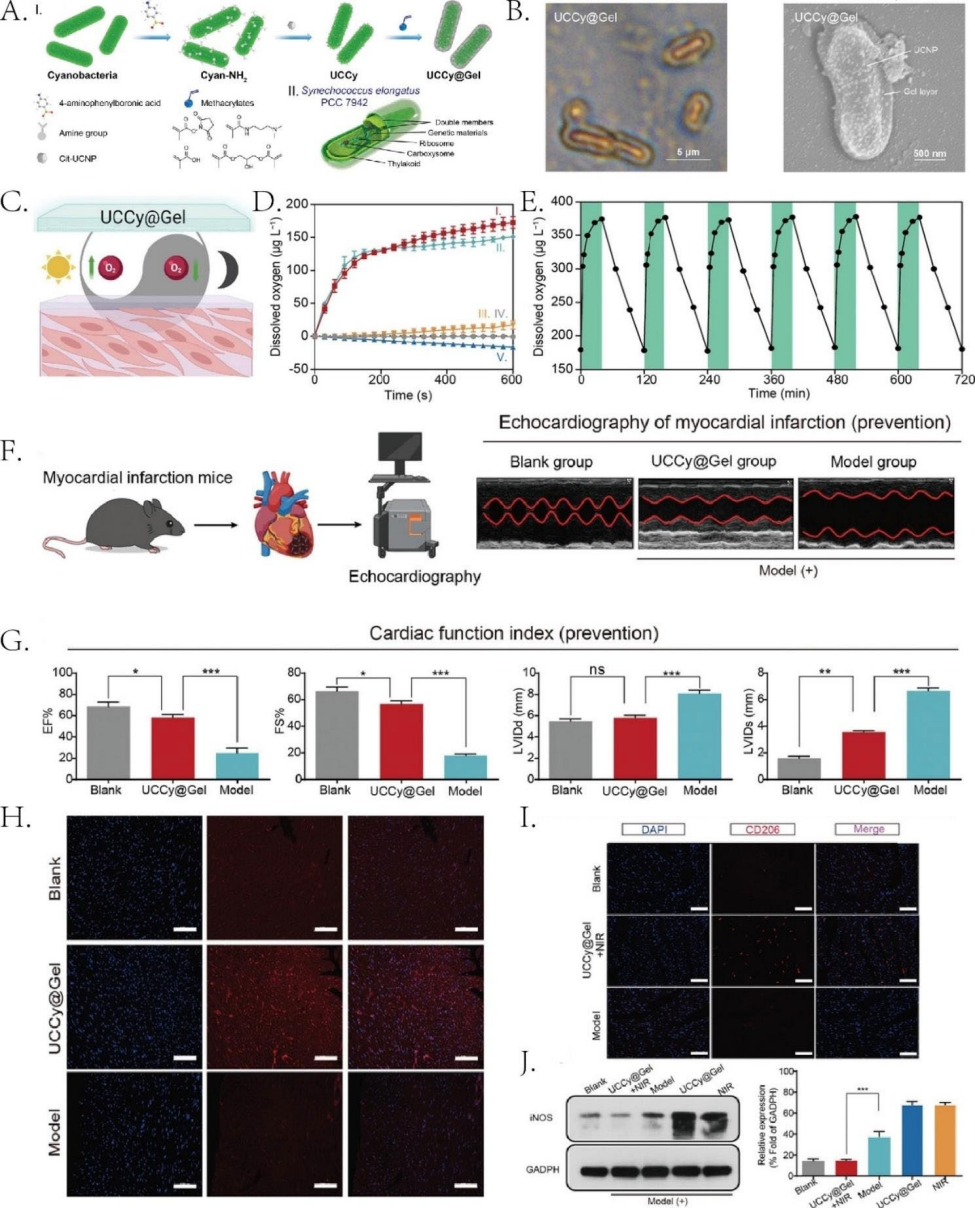
UCCy@Gel functions in MI mice. (**A**) Schematic illustration for the synthesis of UCCy@Gel (I), the schematic illustration for the structure of cyanobacteria (II). (**B**) SEM and TEM images of UCCy@Gel. (**C**) Schematic diagram of photosynthesis and respiration of UCCy@Gel. (**D**) (I) UCCy@Gel + 980 nm NIR light irradiation, (II) UCCy@Gel + white light irradiation, (III) Cyanobacteria + 980 nm NIR light irradiation, (IV) PBS + 980 nm NIR light irradiation and (V) time-oxygen release curves of UCCy@Gel in the dark. (**E**) Activity of UCCy@Gel (green = NIR on; white = dark treatment). (**F**) Schematic representation of dark hypoxia-preconditioned mice and echocardiographic examination of mouse hearts 6 h after modeling and (**G**) assessment of cardiac function by echocardiography. (**H**) Fluorescence staining of HSP70 protein in heat shock after dark hypoxia pretreatment. (**I**) Immunofluorescent staining of CD206 and (**J**) Western blot and quantification of iNOS (M1 macrophage marker) in mouse heart after 980 nm NIR treatment of MI mice for 72 h. Reproduced under terms of the CC-BY license [159]. Copyright 2022, Liu Y, published by Wiley-VCH.

In ischemic tissue, oxygen deficiency is also a major cause of cell death. Hypoxia induces the up-regulation of early growth response 2 (EGR2), leading to the inflammatory response and apoptosis [158]. Therefore, timely oxygen delivery to the ischemic myocardium is an effective way to save ischemic myocardium. Liu et al. [159] designed a nanoparticle loaded with cyanobacteria and encapsulated it in a hydrogel, that is, hydrogel-coated upconversion cyanobacterium nanocapsule (UCCy@Gel). The upconversion nanoparticles (UCNPs) could absorb near-infrared light penetrating deep tissue, producing shorter-wavelength photons. Cyanobacteria can undergo photosynthesis and continuously produce oxygen under its action. In the mouse MI model, cyanobacteria produced oxygen under 980 nm near-infrared light irradiation. The inflammatory response in the infarct area attenuated, the proinflammatory cytokines IL-6 and TNF-α decreased, and the inducible nitric oxide synthase (iNOS) protein down-regulated. When the hydrogel was implanted into the apex of healthy mice and treated in the dark for 2 to 4 h, the cyanobacteria consume oxygen through respiration and form a micro-hypoxic environment locally. Subsequent MI models demonstrated that pre-hypoxic treatment of mice reduced the decline in cardiac function. Hypoxia may be responsible for the up-regulation of HSP70 expression. (Fig. 3)

### Promote angiogenesis

When MI occurs, the ischemic and hypoxic environment may cause hypoxic and inflammatory cells to produce a series of signals, such as vascular endothelial growth factor (VEGF), angiopoietin (ANG), and fibroblast growth factor (FGF). ANG promotes pericyte detachment from blood vessels. They are dissociated from the basement membrane under the action of MMP-mediated proteases. ECM is formed by VEGF-stimulated extravasation of plasma proteins. Endothelial cell permeability is increased due to VEGF stimulation. Endothelial cells migrate to the ECM through the action of integrins. Subsequently, blood vessels tend to mature under the action of VEGF, ANG, and platelet-derived growth factor (PDGF) [160].

Growth factors play a crucial role in new blood vessel formation, but proteases in the body easily degrade them. Therefore, using biomedical materials targets these proangiogenic factors more efficiently in the MI infarct area. Rocker et al. [161] designed a thermoresponsive hydrogel mediated by poly (N-isopropyl acrylamide) (PNIPAM), combining glycol chitosan (GC), PNIPAM, and sulfonate. The hydrogel could deliver VEGF, IL-10, and PDGF sequentially. Injection of hydrogel into the MI mouse model exhibited that functional endothelial cells and pericytes are recruited into the myocardium, and functional angiogenesis was increased. The high loading capacity, sustained and stable cargo release ability, and stimuli responsiveness of the hydrogel enable the delivery of protein molecules. Wu et al. [162] employed a novel temperature-sensitive aliphatic polyester hydrogel to load VEGF, promoting angiogenesis in the MI infarct area. Self-assembling peptide nanofibers can be converted into nanofibrous gels under physiological conditions. This allows it to degrade slowly and release cargo continuously. Therefore, it can serve as a carrier for growth factors. Lin et al. [163] combined VEGF with self-assembling peptide nanofibers. In the porcine MI model, the nanofibers recruited myofibroblasts. VEGF successfully promoted arterial angiogenesis. The basic fibroblast growth factor (bFGF) is the most important in promoting angiogenesis. Garbern et al. [164] polymerized N-isopropylacrylamide (NIPAAm), propylacrylic acid (PAA), and butyl acrylate (BA). Temperature- and pH-responsive injectable hydrogels are fabricated. It turned into a gel at 37 °C and pH 6.8. In the MI rat model, bFGF reaches the infarcted area through the hydrogel. On the seventh day of injection, bFGF retention was 10 times higher than that of the control group. After 28 days, capillary and arteriole density increased by 30–40%. Fu et al. [165] prepared a bFGF-loaded injectable chitosan hydrogel that had similar effects. The hepatocyte growth factor (HGF) can produce proangiogenic effects by activating Met receptors. Guo et al. [166] used self-assembled peptide nanofibers to load hepatocyte growth factor precursor (pro-HGF) activators. It could interact with the β-chain of pro-HGF in the MI infarct to achieve allosteric activation. Nanofibers prolonged the activator’s release time in MI mice and significantly promoted neovascularization in the infarct marginal zone.

In addition to delivering growth factors directly to the ischemic area, the content of growth factors can be increased indirectly to promote angiogenesis. Oncostatin M (OSM) is a cytokine secreted by macrophages. OSM can activate JAK/STAT, MEK, and PI3K-Akt pathways to generate VEGF, thereby promoting angiogenesis [167]. Jiang et al. [168] designed a pH- and temperature-responsive injectable hydrogel-loaded OSM using chitosan (CS), citric acid (CA), and PNIPAM and injected it into MI mice. The hydrogel group has up-regulated VEGF and FGF-2 expressions. Continued induction of growth factor expression by OSM promoted angiogenesis and attenuated ventricular remodeling. The stimulating effect of Bioglass (BG) has unique gene activation properties. According to in vitro studies, BG can stimulate fibroblasts to secrete VEGF and bFGF and promote new blood vessel formation [169]. Qi et al. [170] designed BG-conjugated sodium alginate (SA) injectable hydrogels. The BG-SA hydrogel was injected into the myocardium surrounding the MI infarct area. Immunohistochemistry showed that the expression of VEGF and bFGF were both up-regulated, which successfully promoted the formation of new blood vessels. Bone marrow-derived mesenchymal stromal cells (BM-MSC) have a paracrine function and can secrete VEGF and bFGF. Ciuffreda et al. [171] designed a PEG injectable hydrogel loaded with heparin and BM-MSC. Heparin can capture and release soluble factors (SF). MI rats exhibited VEGF production seven days after hydrogel injection. The VEGF and bFGF production significantly increased after 30 days. Increased number of new blood vessels formed. Cardiac stromal cells also have a similar effect and can promote angiogenesis [172].

Some special components of biomedical materials may play a role in angiogenesis. NO is an unstable molecule produced by the vascular endothelium, which can maintain blood vessel homeostasis. NO synthase (NOS) can convert L-arginine to L-citrulline. NO is released during this process. VEGF can also regulate angiogenesis by increasing NO production [173]. The ROS produced in the MI can oxidize NO to peroxynitrite [174]. Based on these phenomena, Vong et al. [175] designed a composite injectable hydrogel. The b-poly (l-arginine) (PArg) created a triblock copolymer (PArg-PEG-PArg) by cross-linking with PEG. It could produce NO in the body. It was coupled with a redox hydrogel (RIG) to form a novel injectable hydrogel, NO-releasing redox injectable hydrogel (NO-RIG). PEG exhibited a temperature-responsive transformation into a gel in mice. It could regulate the redox balance, scavenge ROS through electrostatic cross-linking and slowly release NO over an extended period. In vivo experiments proved that NO-RIG reduced infarct size and promoted neovascularization. Carbon nanofibers with high surface area, electrical conductivity, and ECM-like support are widely used in MI treatment. Tashakori-Miyanroudi et al. [176] designed a collagen-containing carbon nanofiber (Col-CNF) scaffold. Neovascularization in the infarct region increased while myocardial fibrosis decreased after stenting in MI mice. Carbon nanofibers promote synchronous contraction of the infarcted myocardium, matching the viscoelastic properties of the myocardium.

**Fig. 4:**
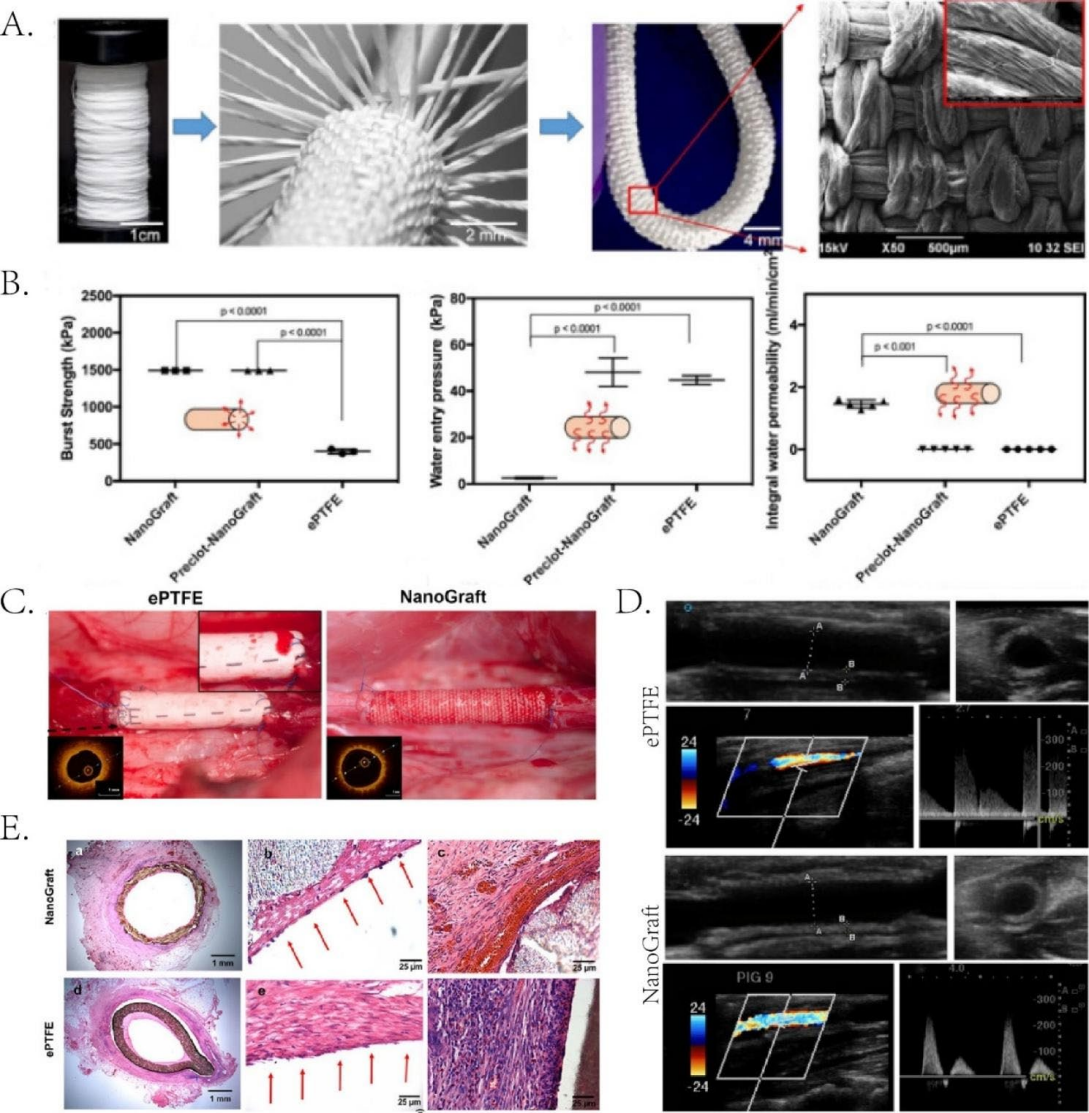
NanoGraft has good biocompatibility in porcine carotid artery transplantation. (**A**) Schematic diagram of NanoGraft preparation (inset shows SEM micrograph). (**B**) Pre-coagulated nanografts show similar physical properties to ePTFE. (**C**) Direct representation of ePTFE graft suture exudation and nanografts without exudation. Insets are OCT images of patent grafts. (**D**) Ultrasound images 4 weeks after implantation of ePTFE and NanoGraft in the porcine carotid artery. (**E**) 2 weeks after implantation (**a**) thrombosis, (**b**) luminal surface and (**c**) inflammatory response of abluminal surface of ePTFE and NanoGraft. Reproduced under terms of the CC-BY license [178]. Copyright 2022, Joseph J, published by BMC

Promoting endogenous angiogenesis through direct or indirect delivery of growth factors or cytokines takes time. Artificial blood vessels can direct blood flow to the infarcted area through a procedure similar to CABG. Tissue Engineered Vascular Grafts (TEVGs) are synthesized from biomedical materials and loaded with bioactive molecules to promote continuous and stable blood flow to the ischemic area [177]. Joseph et al. [178] fabricated a nanofiber vascular graft (NanoGraft) and woven it through strands of nanofibers called yarns. The nanofibers’ porous and physicochemical properties enhanced the graft’s mechanical properties and surface area. Subsequently, a pre-clotting protocol filled the voids of the nanomaterials with fibrin to prevent blood leakage. A vascular graft replaced a segment of the porcine carotid artery. Compared to the implantation of expanded polytetrafluoroethylene grafts (ePTFE), only slight endothelialization of the vessel wall was observed after two weeks of implantation. The inflammatory response was also within an acceptable range. After four weeks, the ultrasound revealed that the NanoGraft remained patent. Additionally, angiogenesis, collagen, elastin, and mucopolysaccharides increased, making grafted vessels more elastic than native vessels. (Fig. 4) This study inspires artificial blood vessel transplantation in MI. Buscemi et al. [95] combined α, β-poly (N-2-hydroxyethyl)-d, l-aspartamide (PHEA), and PLA with PCL to make vascular grafts using electrospinning technology. Low molecular weight heparin was added for anticoagulation. An arteriovenous fistula was established by implanting it into the external iliac vessels of pigs. Despite the tendency to thrombosis after three weeks of implantation, the vascular grafts exhibited good biocompatibility and physicochemical properties. Artificial vascular grafts still have many limitations, such as thrombosis, vascular rupture, and blood extravasation. There is a need to develop artificial blood vessels with good mechanical properties, biocompatibility, and thrombosis resistance. Further in vivo experiments are needed to demonstrate the feasibility of tissue-engineered vascular grafts.

### Improve cardiac function

After MI occurs, dying cardiomyocytes trigger an inflammatory response. A series of growth factors and cytokines lead to the transformation of fibroblasts into myofibroblasts. TGF-β induces the transcription of α-smooth muscle actin (α-SMA) via activating the Smad3 signaling pathway. Therefore, TGF-β inhibition may help alleviate cardiac fibrosis [56]. Additionally, extracellular matrix changes and mechanical stress effects lead to myofibroblast activation. Myofibroblasts have contractile and secretory abilities. They secrete extracellular matrices, such as collagen, to facilitate repair and fibrosis processes, eventually leading to mature scar formation. The structure and capacity of the myocardium at the scar site are severely damaged, leading to heart failure and arrhythmia [179].

Cardiac patches have played an important role in this area. The physical properties of the patch attached to the surface of the heart, such as mechanical support and electrical conductivity, can help the recovery of myocardial function after MI. Furthermore, the cardiac patch can be loaded with stem cells, growth factors, and microRNAs. However, cardiac patches are not always stably attached to the myocardium surface. The uncontrolled release of the payload has also resulted in limited use of the cardiac patch. Cardiac patches developed using biomedical materials largely address these issues [180].

The scar formed in the infarcted area loses its ability to conduct electricity. Therefore, a conductive biomaterial can be implanted to restore the electrical conductivity of this part of the region to relieve arrhythmia. Zhang et al. [181] prepared a conductive hydrogel. Polypyrrole (PPy) was supported on gelatin. These were added to oxidized xanthan gum (OXG) to form injectable hydrogels. PPy is a tissue engineering material with biological stability and high electrical conductivity. When injected into the infarct area of MI rats, myocardial fibrosis tissue’s electrical conductivity and conduction velocity increased significantly. Additionally, ventricular remodeling and the size of the infarct area decreased. The hydrogel exhibited good biocompatibility and electrical conductivity. The electrical conduction has been restored, and arrhythmias have been reduced in the scar area. Song et al. [182] designed a conductive hydrogel cardiac patch using polyacrylic acid (PAA) and oxidized alginate (OA)/gelatin (Geln). This hydrogel exhibited high electrical conductivity, elasticity, compression resistance, and biocompatibility. When implanted in MI rats, good electrical conduction could be achieved. Furthermore, myocardial fibrosis was suppressed four weeks after implantation. Carbon nanomaterials are commonly used conductive materials in tissue engineering. Wang et al. [183] designed conductive cardiac patches using elastin, gelatin, and carbon nanotubes, achieving high mechanical properties, shape memory, and high electrical conductivity. It exhibited improved electrical conduction and cardiac function in rat and mini-pig MI models.

**Fig. 5:**
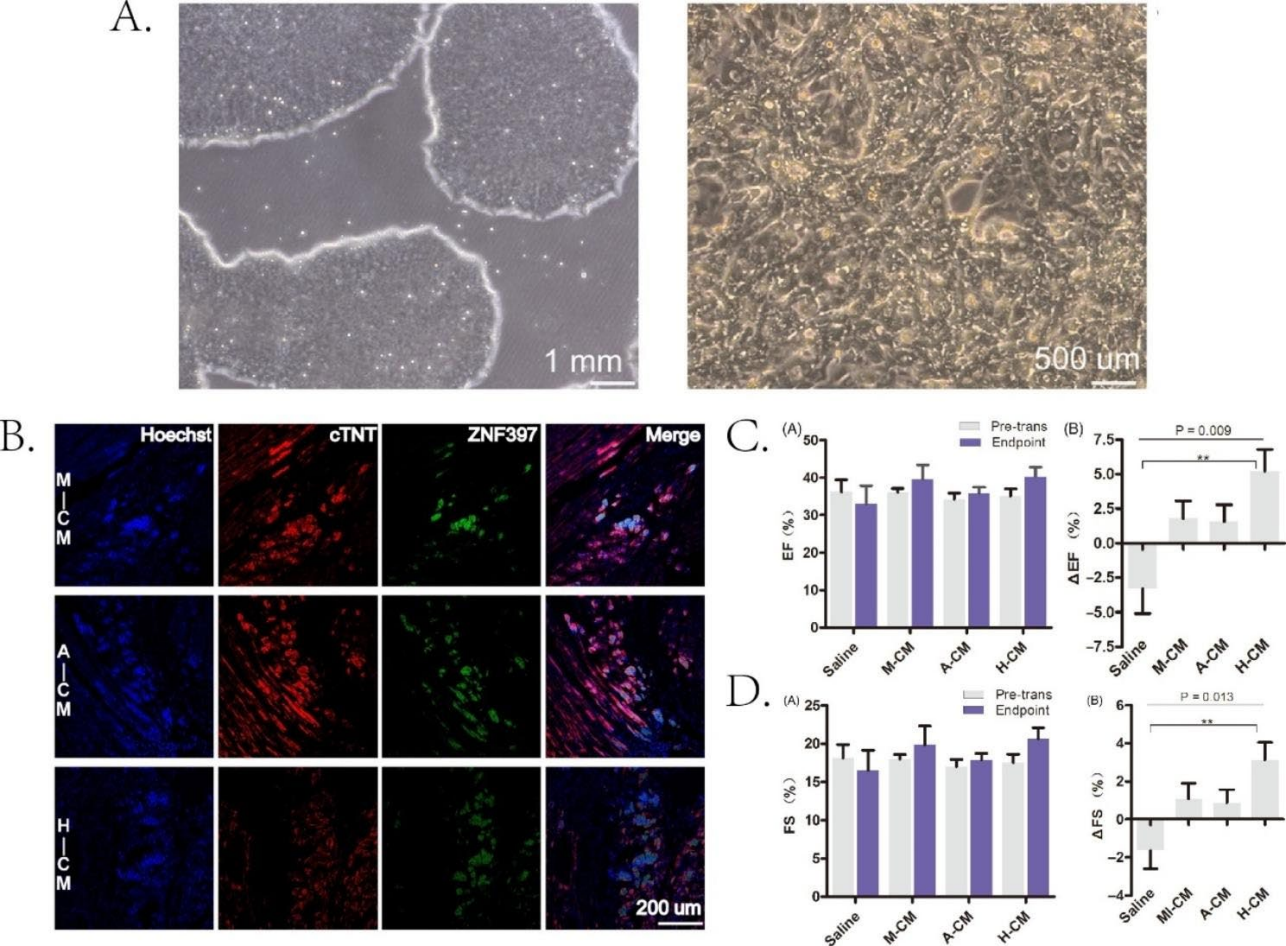
Injectable hydrogel-loaded hESC-CMs promote cardiac regeneration. (**A**) Representative phase contrast image of hESCs before and after CM differentiation. (**B**) Double immunofluorescence staining of human-specific antibody (ZNF397, green) and cTNT (red) of hESC -CM grafts 4 weeks after injection. (**C**) Echocardiographic assessment of the numerical value of EF before and after injection and the percent change in EF of each rat. (**D**) Echocardiographic assessment assessed the numerical value of FS before and after injection and the percent change in FS of each rat. M-CM, matrigel + hESC-CMs; A-CM, alginate + hESC-CMs; H-CM, hyaluronate + hESC-CMs. Reproduced under terms of the CC-BY license [185]. Copyright 2020, Tan Y, published by Wiley-VCH

Allogeneic heart transplantation is the current solution for patients with cardiac insufficiency after MI. However, this treatment technology cannot be carried out in large quantities due to the shortage of donor sources. Therefore, recruiting cells in loss-of-function regions to improve the lost function is a promising solution. Stem cells can differentiate into various cell types, and their cytokines help heart regeneration. Embryonic stem cells are highly differentiated cells that can differentiate into mature cardiomyocytes. However, it cannot be widely used due to ethics, scarcity of sources, and survival rate. Somatic stem cells are abundant, and they are autologous without immune rejection. Therefore, it can be widely used in cardiomyocyte regeneration [184]. Tan et al. [185] prepared injectable hydrogels using HA hydrogels loaded with human embryonic stem cell-derived cardiomyocytes (hESC-CMs). Four weeks after intramyocardial injection of hydrogel in the infarct area of M I rats, the left ventricular ejection fraction (LVEF) increased from 34 to 36% to 40.33 ± 7.41%, and the left ventricular fractional shortening (FS) increased from 17.54 ± 3.28% increased to 20.66 ± 4.30%, attenuating ventricular remodeling. Matrigel and Alginate hydrogels exhibited similar improvement but were weaker than HA hydrogels. (Fig. 5) After stem cell patch implantation, ROS generated by MI leads to stem cell death. Wu et al. [186] developed a prefixed sponge carpet strategy to solve the problem of stem cell cardiac patch cell survival. Wet tissue adhesive hydrogel made from aldehyde dextran sponge (ODS) was loaded with 2-hydroxy-β-cyclodextrin@resveratrol (HP-β-CD@Res). After ODS contacted the myocardium, a Schiff base reaction occurred between the aldehyde group on it and the amino group on the myocardium. Thus, the hydrogel patch closely fits the myocardium. An aqueous hydrazided hyaluronic acid (HHA) containing mesenchymal stem cells (MSCs) was subsequently injected into the ODS hydrogel patch. Aldehyde groups on ODS reacted with hydrazides on HHA to immobilize MSCs on the patch. Res had antioxidant properties. It could clear the ROS in the Infarct area and prevent the death of MSCs. HP-β-CD could induce autophagy to promote myocardial repair. The encapsulation of Res in HP-β-CD could improve its water solubility and bioavailability. In the MI rat model, cardiac function improved 28 days after surgery. LVEF and FS were 73.70 ± 5.23% and 43.52 ± 4.74%, respectively. The fibrosis area was also reduced to 8.2 ± 1.25%. Left ventricular thickness reached 1.45 mm ± 0.15 mm. MSCs were still alive 21 days after transplantation. The application of injectable collagen scaffolds also promoted the long-term retention of MSCs in the infarct area [187]. Additionally, high-oxygen hydrogels can release O_2_. MSCs can survive and proliferate in hyperoxic hydrogels [188]. They may be used as cell carriers for cell transplantation to improve survival. Similar to embryonic stem cells, induced pluripotent stem cells (iPSCs) have the potential to differentiate into mature myocardium. In contrast to embryonic stem cells, iPSCs have fewer ethical constraints. Consequently, it has a wide range of application potential. Chow et al. [189] loaded human induced pluripotent stem cell-derived cardiomyocytes (iPSC-CM) and erythropoietin (EPO) on PEG hydrogels. The muscle fibers in the infarct area and the ejection fraction significantly increased 10 weeks after implantation in the MI rat model. This may provide ideas for chronic MI repair. This study did not observe any preservation of iPSC-CMs in rat myocardial tissue.

Heart tissue contains its stem cells, called pluripotent cardiac stem /progenitor cells (CSCs). These cells can regenerate into cardiomyocytes under active stimulation. CSCs expressing stem cell markers can be induced, such as c-kit and Sca-1 [190]. Phosphorylated 7-amino-acid peptide (7AP) can promote stem cell migration, proliferation, and differentiation. Zhang et al. [62] loaded 7AP in collagen I hydrogels and injected them into MI mice. LVEF, infarct size, and left ventricular end-diastolic diameter (LVEDd) improved 14 days after surgery. The infarct wall thickness increased, and new blood vessels also appeared in the infarcted area. Moreover, immunohistochemistry revealed that Sca-1 cardiac stem cells appear in the infarcted area. 7AP could stimulate cardiomyocytes to initiate the cell cycle and increase proliferation. Decellularized ECM is an excellent scaffold. It has a similar composition to ECM and can mimic the natural extracellular environment. Singelyn et al. [191] designed a cardiomyocyte extracellular matrix injectable hydrogel derived from the porcine ventricle. Porous nanofibrous scaffolds could be formed in vivo. Two weeks after rat MI, the hydrogel increased native Ki67-positive cardiomyocyte accumulation in the infarct area, with a small amount of c-kit cardiac progenitors. Additionally, cardiac function was improved, and arrhythmias were reduced.

The miRNA can regulate downstream signaling by activating or inhibiting the mRNA expression, affecting cellular activity, but miRNA is easily degraded in the body. Biomedical materials can improve miRNA stability. Yang et al. [192] designed a kind of Carbon dots (CDs), modified with branched polyethyleneimine and loaded with miRNA via electrostatic interactions. CDs had stability, high water solubility, and low toxicity, which could prevent miRNA degradation. In vitro cell culture, nanocomplexes promote the transformation of fibroblasts into inducible cardiomyocytes, as demonstrated by increased transcription of specific mRNA. Nanocomplexes were injected in MI mice. Infarct and scar size were significantly reduced after four weeks. The miR-199a-3p can stimulate HOMER1, CLIC5, and caveolin-2 to promote cardiomyocyte and endothelial cell proliferation. Yang et al. [193] utilized nanoparticle-loaded injectable hydrogels to deliver miR-199a-3p. In MI rats, LVEF increased from 45 to 64% after four weeks of treatment. Post-infarction scar area was reduced from 20 to 10%. The blood vessels in the infarct margin increased significantly.

## Conclusion and prospect

MI is a cardiovascular emergency that can lead to heart failure, arrhythmias, and sudden death. Prompt diagnosis is essential to take early steps to restore the blood supply to the ischemic myocardium. cTns is the gold standard for MI diagnosis, and the current detection methods are time-consuming and require a large sample size. Furthermore, the rapid metabolism and lack of targeting of contrast agents limit imaging diagnosis. Reperfusion is the preferred treatment for MI. However, existing treatments can only restore blood flow and have no effective control measures for early inflammatory and immune responses. There is also a lack of effective intervention methods for angiogenesis in the infarcted area and the recovery of dead myocardial function.

Biomedical materials are widely used in MI research and have great potential. Biomedical materials, such as cardiac patches, hydrogels, nanomaterials, and artificial blood vessels, offer more promise. The biomaterial application shortens the detection time of cardiac markers to minutes and significantly reduces the detection concentration. The hydrogel has a high surface area and porous structure, allowing it to adsorb more cardiac markers. The high stability and signal amplification effect of nanomaterials increases the sensitivity and specificity of cardiac marker detection. Biomedical materials also offer unique advantages in the imaging field. Metal nanomaterials can target the infarct area, and their high stability and magnetic properties play an important role in MRI diagnosis. Additionally, the high permeability of nanomaterials has a greater effect than contrast agents in CT diagnosis, and the controllable degradation rate extends the half-life. Biomedical materials offer more avenues for MI treatment. They can be loaded with cargoes, such as drugs, cytokines, miRNAs, and cells. Stimuli-responsiveness and targeting allow targeted delivery and controlled cargo release, avoiding degradation and improving therapeutic efficiency. They also act as scaffolds to support the proliferation of cells and matrices and blood flow. The physical and chemical properties of biomedical materials also have certain therapeutic effects.

Recently, the advent of microfluidics has expanded the possibilities for biomedical materials. Microfluidics can manufacture highly reproducible nanoparticles by controlling their size, structure, composition, and shape [194, 195]. Microfluidics’ high integration capabilities allow them to detect small samples. Song et al. [196] applied microfluidics to detect myocardial markers CK-MB, Mb, and cTnI, requiring only 1 µL of serum isolated from finger blood. The small volume of microfluidics makes point-of-care detection of myocardial markers promising. 3D printing technology has also facilitated the manufacture of biomedical materials. Noor et al. [197] induced redifferentiation of the patient’s omentum tissue cells into cardiomyocytes and endothelial cells, combined with decellularized ECM as bioinks. A functional vascularized cardiac patch was designed using 3D printing technology with good biocompatibility and matching anatomical properties. Live cell therapy has limitations, such as tumorigenicity, immunogenicity, and low survival, and synthetic cells are an effective alternative. Synthetic cells are membrane-like vesicles containing various acting molecules that can fully mimic cell activity. Their production can also be combined with microfluidics for convenient and fast application [198].

Biomedical materials have significant advantages over traditional methods. However, many challenges remain in clinical translation, such as biocompatibility, immune response, degradation rate, degradation product toxicity, and thrombosis induction. Several issues need to be addressed before clinical application. In the detection of myocardial markers, most tests are achieved by immunofluorescence. It depends on antigen-antibody binding. It is required that the biomaterial does not produce additional antigenicity. So as not to interfere with the antigen-antibody response of the target sample. Second, the test results need to be intuitive, quantifiable, stable, and low-cost. As for imaging diagnosis, biomedical materials are injected into the body as contrast agents. Therefore, it is necessary to determine the most suitable injection dose and ensure its high safety. In addition, it must be able to be rapidly metabolized as a contrast agent. The scan time should also be minimized to prevent the accumulation of contrast material in the body. Biomedical materials should be targeted to achieve high-concentration aggregation in the target organ to improve their temporal and spatial resolution. In terms of treatment, the occurrence of foreign body reactions after the implantation of biological materials may bring damage to the recipient. Therefore, further in vivo studies are needed to rule out potential pitfalls. The method of implantation also needs to be fully considered. As far as possible, biomedical materials should be implanted by non-invasive methods such as intravenous injection rather than puncture surgery, because the injury caused by surgery is not conducive to the recovery of patients. In addition, biomedical materials that act by local injection should have a firm adhesion that prevents their translocation to non-therapeutic areas to cause harm. Secondly, the degradation rate of biomedical materials needs to be effectively controlled. Their degradation rate should not be too fast to guarantee the sustained therapeutic effect of the loaded bioactive substances. The toxicity of degradation products is also of concern. Artificial blood vessel transplantation is more stringent. High vascular endothelial coverage, prevention of thrombosis, and high biocompatibility limit its application [199, 200]. Therefore, a large number of in vivo studies are still needed to ensure the safety and efficacy of biomedical materials and to address the limitations of biomedical materials’ application in vivo.

This review focuses on the application progress of different biomedical materials for MI diagnosis and treatment. The biomaterial application improves the accuracy and efficiency of MI diagnosis. Regarding therapy, they offer further possibilities for reducing inflammation, immunomodulation, inhibiting fibrosis, and cardiac regeneration. Biomedical materials are promising tools that enable new clinical diagnosis and treatment systems, although they have some limits in vivo applications. In the future, researchers from various fields and disciplines are still needed to develop more functional biomedical materials and truly transform biomedical materials from preclinical research to clinical applications, improving the quality of life and saving the lives of more MI patients by taking advantage of biomedical materials in MI diagnosis and treatment.

## Data Availability

Not applicable.

## Abbreviations

MI: Myocardial infarction
CVD: Cardiovascular disease
ECG: Electrocardiogram
STEMI: ST-segment elevation myocardial infarction
NSTEMI: Non-ST-segment elevation myocardial infarction
tPA: Tissue plasminogen activator
PCI: Percutaneous coronary intervention
CABG: Coronary artery bypass grafting
DAMPs: Danger-associated molecular patterns
IL: Interleukin
PGS: Polyglycerol sebacate
PCL: Polycaprolactone
PEG: Polyethylene glycol
PLA: Polylactic acid
PVA: Polyvinyl alcohol
PVP: Polyvinyl pyrrolidone
dECM: Decellularized extracellular matrix
PAM: Polyacrylamide
ECM: Extracellular matrix
PET: Polyethylene terephthalate
PCU: Polycarbonate polyurethane
TPU: Thermoplastic polyurethane
PLGA: Poly lactic-co-glycolic acid
Mb: Myoglobin
h-FABP: Heart-type fatty acid-binding protein
cTns: Cardiac troponins
CK: Creatine kinase
CK-MB: Creatine kinase isoenzyme
BNP: B-Type natriuretic peptide
GSH: Glutathione
LDH: Lactate dehydrogenase
CRP: C reactive protein
AST: Aspartate transaminase
ELISA: Enzyme-linked immunosorbent assay
ECL: Electrochemiluminescence
SPR: Surface plasmon resonance
PEC: Photoelectrochemical
POCT: Point-of-care test
LOD: Limit of detection
CL: Chemiluminescence
NT-proBNP: N-terminal pro-B-type natriuretic peptide
MRI: Magnetic resonance imaging
CT: Computed tomography
US: Ultrasound
FMT: Fluorescence molecular tomography
PET: Positron emission tomography
HMGB1: High mobility group protein B1
RAGE: Receptors for advanced glycation end products
TLR: Toll-like receptor
NF-κB: Nuclear factor kappa B
ERK: Extracellular signal-regulated kinases
IR: Ischemia-reperfusion
ROS: Reactive oxygen species
eNABs: Engineered neutrophil apoptotic bodies
EGR2: Early growth response 2
iNOS: Inducible nitric oxide synthase
VEGF: Vascular endothelial growth factor
ANG: Angiopoietin
FGF: Fibroblast growth factor
PDGF: Platelet-derived growth factor
bFGF: Basic fibroblast growth factor
HGF: Hepatocyte growth factor
OSM: Oncostatin M
BG: Bioglass
NOS: NO synthase
TEVGs: Tissue Engineered Vascular Grafts
α-SMA: α-smooth muscle actin
LVEF: Left ventricular ejection fraction
FS: Fractional shortening
ODS: Aldehyde dextran sponge
MSCs: Mesenchymal stem cells
iPSCs: Induced pluripotent stem cells
LVEDd: Left ventricular end-diastolic diameter
CDs: Carbon dots
